# Pattern Formation under Deep Supercooling by Classical Density Functional-Based Approach

**DOI:** 10.3390/e25050708

**Published:** 2023-04-24

**Authors:** Kun Wang, Wenjin Chen, Shifang Xiao, Jun Chen, Wangyu Hu

**Affiliations:** 1 College of Materials Science and Engineering, Hunan University, Changsha 410082, China; 2 Department of Applied Physics, Hunan University, Changsha 410082, China; 3 Institute of Applied Physics and Computational Mathematics, Beijing 100088, China

**Keywords:** phase field crystal, classical density functional theory, crystalization, supercooling

## Abstract

Solidification patterns during nonequilibrium crystallization are among the most important microstructures in the natural and technical realms. In this work, we investigate the crystal growth in deeply supercooled liquid using the classical density functional-based approaches. Our result shows that the complex amplitude expanded phase-field crystal (APFC) model containing the vacancy nonequilibrium effects proposed by us could naturally reproduce the growth front nucleation (GFN) and various nonequilibrium patterns, including the faceted growth, spherulite, symmetric and nonsymmetric dendrites among others, at the atom level. Moreover, an extraordinary microscopic columnar-to-equiaxed transition is uncovered, which is found to depend on the seed spacing and distribution. Such a phenomenon could be attributed to the combined effects of the long-wave and short-wave elastic interactions. Particularly, the columnar growth could also be predicted by an APFC model containing inertia effects, but the lattice defect type in the growing crystal is different due to the different types of short-wave interactions. Two stages are identified during the crystal growth under different undercooling, corresponding to diffusion-controlled growth and GFN-dominated growth, respectively. However, compared with the second stage, the first stage becomes too short to be noticed under the high undercooling. The distinct feature of the second stage is the dramatic increments of lattice defects, which explains the amorphous nucleation precursor in the supercooled liquid. The transition time between the two stages at different undercooling is investigated. Crystal growth of BCC structure further confirms our conclusions.

## 1. Introduction

Nonequilibrium crystallization in supercooled liquid is frequently encountered in the natural (e.g., snowflakes and minerals) and technical realms, for example, the dendrites in traditional as-cast materials or additively manufactured parts [1] and spherulites in Se [2], polymers [3] and so on. In many cases, supercooling arises from a sudden change in environmental variations, such as in temperature or pressure, for example, quick quenching under liquid nitrogen and solidification under strong shock compressions. Spatially heterogeneous dynamics in the supercooled liquid have been well established experimentally [4,5], which have numerous consequences on the transport properties. The most important transport properties relevant to the crystallization are the shear viscosity and the molecular mobilities determined by the translational and rotational diffusion coefficients [6]. The latter ones directly control detailed manners of how molecules attach and align with the growing crystal. Particularly, the spherulitic growth patterns are the results of competition between the translational and rotational motions, which have ever puzzled researchers in the related field for a long time [3,7] and later were clarified by Gránásy et al. [8,9] using the phase-field model extended via allowing for evolutions of local crystal orientations, termed the orientation field-based phase-field (OFPF) model. A key result of the competition is the emergence of growth front nucleation (GFN), which is a new formation mechanism for polycrystalline formations in contrast to the traditional one through impinging among growing single crystals [10]. Despite the great achievements in the understanding of nonequilibrium crystallization, present knowledge based on the OFPF model relies on the assumption that crystals are free to change their local orientation to lower the free energy, which is not entirely true for crystals and sometimes results in predictions that are qualitatively wrong. For example, the rotation and shrinking of circular grain embedded into an infinite crystal cannot be correctly predicted by the OFPF model due to geometrical constraints on the dislocations (See the review paper [10]). This indicates that microstructures at the atom level are important. Indeed, Tegze et al. [11] found that the diffusion-controlled (or slow) growth mode and the diffusionless (or fast) steady growth mode during equilibrium crystallizations have distinctly different interface structures. The interface for the former is fairly thin and faceted, while the latter extends several atomic layers towards the liquid, leaves a so-called crystalline state in the liquid and shows rounded corners. As a result, different growth morphology emerges. Actually, the evolution of the local crystal orientation, the interface energy and its anisotropy are governed by atomic structures and the latter is affected by external conditions, such as the undercooling of the solidification system, among others. Thereby, a complete understanding of nonequilibrium crystallization requires us to treat the crystal growth at a more fundamental level, i.e., in terms of the atomic structures, as well as its kinetics. However, simulation of the crystallization process by present atomic simulation methods, such as classical molecular dynamics, remains a great challenge at present due to the limitation of spatial and temporal scale. 

The classical density functional theory-based phase-field crystal (PFC) model, first proposed by Elder et al. [12], is a promising candidate and has been used to predict the symbiosis of faceting with growth mode selection via appending a colored Gaussian noise term in the equation of motion for evolutions of the reduced atom density [11]. Further, the morphological transition of nonequilibrium crystal growth is studied in depth by others [11,13,14,15]. Moreover, the amorphous nucleation precursor in the supercooled liquid is successfully caught by the PFC model [16]. Despite these progresses made by the PFC model, the crystallization behaviors are only discussed in the solid–liquid coexistence region of the PFC phase diagram, corresponding to the small supercooling. The crystallization pattern under deep supercooling is not studied at present. It is known that the vacancy concentration in crystals under deep supercooling deviates much farther from its equilibrium state than the one under small supercooling. As a result, the solidification microstructures at the atom level, such as dislocation density in the growing grains, may be considerably altered due to the nonequilibrium vacancy concentration (NEVC) effects. In addition, the elastic interactions, including both long-wave and short-wave components, are important for nonequilibrium crystallization under deep supercooling. However, the original PFC model only describes the long-wave elastic interactions or the slow dynamics (low-frequency events) [17] and the microstructure evolutions near the thermodynamic equilibrium states. Phenomenologically, fast dynamics carrying the short-wave interaction have been described using a memory kernel [18]. In particular, when the memory kernel takes the form of the exponential relaxation, i.e., Maxwell memory function, the motion equation for the PFC model becomes a hyperbolic partial differential equation, which coincides with the modified PFC model proposed by Stefanovic et al. [17] and could describe the elastic interactions that are missing in the original PFC model. Recently, Podmaniczky et al. [19] successfully grasped the GFN as well as the spherulite in the undercooled system using a hydrodynamics-coupled phase-field crystal (HPFC) model. Although not all the nonequilibrium phenomena during the crystallizations, such as the dendritic growth among others, are shown in their results, it implies that the fast dynamics or the short-wave interaction contained in the hydrodynamics, but absent in the PFC model, is crucial for describing the nonequilibrium phenomenon during crystallization. As a faster version of the PFC model, the amplitude-expanded PFC (APFC) model first derived by Goldenfeld et al. [20,21] should be more suitable for the nonequilibrium crystallizations since the simulated system could be much larger and thus allow the morphology of a growing grain to develop for sufficient time. Additionally, the elastic interactions missing in the APFC model has been incorporated by coarse-graining the hyperbolic PFC model, which results in a hyperbolic APFC model [22]. However, it is peculiar that such model is not applied to simulate the frequently encountered nonequilibrium crystallization patterns, such as dendrite, spherulite and so on, albeit with a few theoretical analyses [22,23,24]. 

In this work, the crystallization behaviors of a model crystal, i.e., the hexagonal crystal, under deep supercooling are explored using a modified APFC model containing the NEVC effects recently proposed by us [25] and the hyperbolic APFC model, respectively. Through comparative investigations, the roles of the short-wave interactions due to the inertia effects and the NEVC effects on the nonequilibrium crystallization behaviors are examined. In particular, the seed spacing and distribution on the pattern formation are investigated using the modified APFC model, which receives little attention in literatures. Our results uncover that the seed spacing and distribution have a significant influence on the solidification patterns, as well as the atomic structures under deep supercooling. This paper is organized as follows. This part introduces the background and motivations of this work. Then, in Section 2, three simulation methods based on the classical density functional theory are introduced. Next, in Section 3, the nonequilibrium solidifications of hexagonal crystals are investigated by the aforementioned simulation methods. Further, in Section 4, the results obtained from the hexagonal crystals are examined in other crystals, taking the BCC crystal as an example. Finally, the conclusion is arrived at in Section 5. In this work, if not specified, we use bold letters to denote vector or tensor and the corresponding italicized letter with certain subscripts (represented by Greek letters, such as α,β,…) to denote its Cartesian component.

## 2. Classical Density Functional Theory-Based APFC Model and Its Phenomenological Corrections

### 2.1. Original APFC Model

In classical density functional theory [26,27], Helmholtz free energy (ℱ) of the solid–liquid coexistent system could be expressed as a summation of the noninteractive part $\left({ℱ}_{id}\right)$, excess free energy $\left({ℱ}_{ex}\right)$ and the contribution from external fields, which is a functional of one-particle density (ρ(r)). Particularly, ${ℱ}_{id}$ is, in essence, the free energy of ideal gas, i.e.,

(1) $${ℱ}_{id}=k_{B}T\int^{\;}\rho \left[\ln\left(\Lambda^{d}\rho \right)-1\right]dr,$$

where $k_{B}$ is the Boltzmann constant, T is temperature, d is the dimension of the system and Λ is the thermal de Broglie wavelength. The exact expression for ${ℱ}_{ex}$ depends on the detailed interactions of the system, which is usually unknown a priori. Under the Ramakrishnan–Yussouff approximation [27], the excess free energy for pure elements can be expressed by

(2) $${ℱ}_{ex}\left(T,\left[\rho \left(r\right)\right]\right)=-\frac{1}{2}k_{B}T\int^{\;}\int^{\;}dr_{1}dr_{2}\Delta \rho \left(r_{1}\right)C^{\left(2\right)}\left(r_{1},r_{2}\right)\Delta \rho \left(r_{2}\right),$$

where $C^{\left(2\right)}$ is a direct pair correlation function and $\Delta \rho \left(r_{j}\right)=\rho \left(r\right)-\rho_{l}$, $\rho_{l}$ is the number density of the liquid phase. The PFC model further estimates $C^{\left(2\right)}$ by gradient expansions to the fourth order [26], i.e.,

(3) $$C^{\left(2\right)}\approx C_{0}^{\left(2\right)}-C_{2}^{\left(2\right)}\nabla_{r}^{2}+C_{4}^{\left(2\right)}\nabla_{r}^{4},$$

where $C_{k}^{\left(2\right)}$ (*k* = 0, 2, 4) are expansion coefficients, and the gradients of odd order vanish due to $C^{\left(2\right)}\left(r\right)=C^{\left(2\right)}\left(-r\right)$. The dimensionless reduced density is defined as follows:

(4) $$\psi \left(r\right)=\left(\rho \left(r\right)-\bar{\rho }\right)/\bar{\rho },$$

where $\bar{\rho }$ is the average number density. Through expanding the integrand of ${ℱ}_{id}$ around ψ=0 and combining with Equations (2) and (3), the free energy as a function of ψ(r) is obtained. Various variants can be formulated, but they differ only in the coefficients preceding each power term of ψ(r). The Swift–Hohenberg model [12,28] is one of the variants, whose dimensionless free energy functional reads

(5) $$ℱ=\int^{\;}dx\left\{\frac{\psi }{2}\left[-\epsilon +{\left(1+\nabla^{2}\right)}^{2}\right]\psi +\frac{\psi^{4}}{4}\right\},$$

where ϵ is a small quantity proportional to the undercooling extent of the system. Fourier expansion of ψ, to the principal reciprocal lattice vectors (RLVs) of the considered structure, gives

(6) $$\psi \left(x\right)=\bar{\psi }+\overset{N}{\underset{j=1}{\sum }}\eta_{j}\exp\left(iK^{\left(j\right)}\cdot x\right)+c.c.,$$

where c.c. denotes the complex conjugate of the summation term, $\bar{\psi }$ is average reduced density and $\eta_{j}$ is the j-th complex amplitude corresponding to the reciprocal lattice vector (RLV) $K^{\left(j\right)}$. The summation runs over all (totally *N*) principal RLVs. Hereafter, we will take 2D hexagonal lattices and 3D BCC lattices as an example. For hexagonal lattices, the RLVs are

(7) $$K^{\left(1\right)}=k_{0}\left(-\sqrt{3}/2,-1/2\right),\;K^{\left(2\right)}=k_{0}\left(0,1\right),\;K^{\left(3\right)}=k_{0}\left(\sqrt{3}/2,-1/2\right),$$

where $k_{0}=1$ at equilibrium (reference) states. They satisfy a triadic resonance condition of $\sum_{j=1}^{3}K^{\left(j\right)}=0$. For BCC lattices, the RLVs are

(8) $$K^{\left(1\right)}=k_{0}\left(1,1,0\right),\;K^{\left(2\right)}=k_{0}\left(1,0,1\right),\;\;K^{\left(3\right)}=k_{0}\left(0,1,1\right),\;\;K^{\left(4\right)}=k_{0}\left(0,1,-1\right),\;K^{\left(5\right)}=k_{0}\left(1,-1,0\right),\;K^{\left(6\right)}=k_{0}\left(-1,0,1\right).$$

where $k_{0}=\sqrt{2}/2$. Note that the following triadic and quartic resonances are satisfied, i.e.,

(9) $$K^{\left(1\right)}-K^{\left(4\right)}-K^{\left(2\right)}=0,\;K^{\left(1\right)}+K^{\left(6\right)}-K^{\left(3\right)}=0,\;\;K^{\left(4\right)}+K^{\left(5\right)}+K^{\left(6\right)}=0,\;K^{\left(2\right)}-K^{\left(5\right)}-K^{\left(3\right)}=0,$$

and

(10) $$K^{\left(1\right)}-K^{\left(3\right)}-K^{\left(4\right)}-K^{\left(5\right)}=0,\;K^{\left(1\right)}-K^{\left(2\right)}+K^{\left(5\right)}+K^{\left(6\right)}=0,\;K^{\left(2\right)}-K^{\left(3\right)}+K^{\left(4\right)}+K^{\left(6\right)}=0.$$

We proceed by following the variational approach [29,30] to derive the coarse-grained model. By substituting Equations (6) and (7) into the integrand of Equation (5), integrating the result over a unit cell and applying the corresponding resonance conditions, the coarse-grained free energy density can be obtained by dividing the result by the cell volume. Because the average density and amplitudes vary slowly in space in comparison with the density wave itself, $\bar{\psi }$ and $\eta_{j}$ can be viewed as functions of a slowly varying spatial variable and thus remain constant during cell integration. After long but straightforward algebra calculations, the resulting coarse-grained free energy functional is

(11) $${ℱ}^{cg}=\int^{\;}dx\left\{\frac{1}{2}\left(-\epsilon +1\right){\bar{\psi }}^{2}-g\frac{{\bar{\psi }}^{3}}{6}+\lambda \frac{{\bar{\psi }}^{4}}{12}-\left(\epsilon +g\bar{\psi }-\lambda {\bar{\psi }}^{2}\right)A^{2}+\lambda A^{4}-\frac{1}{2}\lambda \sum_{j=1}^{N}{\left|\eta_{j}\right|}^{4}+f_{s}\left(\bar{\psi },\eta_{j},\eta_{j}^{*}\right)+\overset{N}{\underset{j=1}{\sum }}{\left|G_{j}\eta_{j}\right|}^{2}\right\}$$

where $G_{j}=\nabla^{2}+2iK^{\left(j\right)}\cdot \nabla$, $A^{2}=\sum_{j=1}^{N}{\left|\eta_{j}\right|}^{2}$ and $f_{s}$ is a structure-dependent function. In addition, the higher-order derivative terms of $\bar{\psi }$ have been dropped since these terms would lead to numerical instabilities [29] and can be self-consistently eliminated through a convolution operator [31]. The structure-dependent functions for hexagonal lattices and BCC lattices are

(12) $$f^{s}\left(\bar{\psi },\eta_{j},\eta_{j}^{*}\right)=-\left(g-2\lambda \bar{\psi }\right)\left(\prod_{j=1}^{N}\eta_{j}+\prod_{j=1}^{N}\eta_{j}^{*}\right)$$

and

(13) $$f^{s}\left(\bar{\psi },{\hat{\eta }}_{j},{\hat{\eta }}_{j}^{*}\right)=-\left(g-2\lambda \bar{\psi }\right)\left(\eta_{1}^{*}\eta_{2}\eta_{4}+\eta_{3}^{*}\eta_{1}\eta_{6}+\eta_{4}\eta_{5}\eta_{6}+\eta_{2}^{*}\eta_{3}\eta_{5}+c.c.\right)\;+2\lambda \left(\eta_{1}^{*}\eta_{3}\eta_{4}\eta_{5}+\eta_{2}^{*}\eta_{1}\eta_{5}\eta_{6}+\eta_{3}^{*}\eta_{2}\eta_{4}\eta_{6}+c.c.\right),$$

respectively. 

The complex amplitudes obey nonconserved dissipative dynamics, while the average density follows conserved dissipative dynamics, i.e.,

(14) $$\frac{\partial \eta_{j}}{\partial t}=-M_{\eta_{j}}\frac{\delta {ℱ}^{cg}}{\delta \eta_{j}^{*}}+\varsigma_{\eta_{j}},\;\left(j=1,2,3\right)$$

(15) $$\frac{\partial \bar{\psi }}{\partial t}=\nabla \cdot \nabla \left(M_{\bar{\psi }}\frac{\delta {ℱ}^{cg}}{\delta \bar{\psi }}\right)+\nabla \cdot \varsigma_{\bar{\psi }},$$

where $M_{\eta_{j}}$ and $M_{\bar{\psi }}$ represent mobility parameters of the amplitude $\eta_{j}$ and $\bar{\psi }$, respectively, and are taken to be a unit in the present work. In addition, coarse-grained thermal fluctuations for $\eta_{j}$ and $\bar{\psi }$ can be considered by appending the stochastic variables $\varsigma_{\eta_{j}}$ or $\varsigma_{\bar{\psi }}$ to the right hand of Equation (14) or (15), which are not the concern in this work. It is known that the motion equations given by Equations (14) and (15) only describe the overdamped dynamics and the elastic interactions are missing. 

### 2.2. Hyperbolic APFC Model

According to the idea of Galenko et al. [18,22], the fast time scale due to the elastic interactions could be introduced into the APFC model by coarse-graining the hyperbolic PFC model [18] or the modified PFC model [17], which gives

(16) $$\tau \frac{\partial^{2}\eta_{j}}{\partial t^{2}}+\frac{\partial \eta_{j}}{\partial t}=-M_{\eta_{j}}\frac{\delta {ℱ}^{cg}}{\delta \eta_{j}^{*}},\;\left(j=1,2,\ldots ,N\right)$$

To accelerate our calculations, we use $G_{j}\approx 2iK_{j}\cdot \nabla$, which has been widely applied during theoretical analyses of the solid–liquid interface, for example, in the studies [22,23]. By inserting Equation (11) into the above equation and rearranging the resulting terms according to the degree of $\eta_{j}$’s, we get

(17) $$\tau \frac{\partial^{2}\eta_{j}}{\partial t^{2}}+\frac{\partial \eta_{j}}{\partial t}=-M_{\eta_{j}}\left[-\left(\epsilon +g\bar{\psi }-\lambda {\bar{\psi }}^{2}\right)\eta_{j}+2\lambda \overset{N}{\underset{k=1}{\sum }}{\tilde{c}}_{jk}\eta_{j}{\left|\eta_{k}\right|}^{2}+f_{s,\eta_{j}^{*}}^{’}-{\left(2K^{\left(j\right)}\cdot \nabla \right)}^{2}\eta_{j}\right]\;\;\;\;\;\;\;\;\;\;\;\;\;\;\;\;\;\;\;\;\;\;\;\;\;\;\;\;\;\;\;\;\;\;\;\;\;\;\;\;\;\;\;\;\;\;\;\;\;\;\;\;\;\;\;\;(j=1,2,\ldots ,N)$$

where

(18) $${\tilde{c}}_{jk}=\{\begin{array}{c} 1,\;\;\left(j\neq k\right)\\ \frac{1}{2},\;\;\left(j=k\right) \end{array}$$

In the above equation, we used ${\left(\cdot \right)}_{,X}^{’}$ to represent the first derivative of the function (·) with respect to X in this work, for example, $f_{s,\eta_{j}^{*}}^{’}\equiv \partial f_{s}/\partial \eta_{j}^{*}$. Through Floquet stability analysis [17], it is found that the effective vacancy diffusion coefficient is proportional to $M_{\eta_{j}}$ and the elastic interaction length is proportional to $\sqrt{\tau M_{\eta_{j}}}$. Thereby, the elastic interaction length can be tuned by τ so as to adapt elastic wave modes typically on time scales many orders of magnitude slower than phonon vibrations but still much faster than diffusive time scales. This facilitates us to investigate the influence of different short-wave interactions on the nonequilibrium solidification behaviors in this work.

### 2.3. Modified APFC Model Containing the NEVC Effects

Dislocation is the most fundamental lattice defect that the APFC model can describe. For 2D systems, only edge dislocations can be caught. The osmotic force due to the NEVC tends to make the dislocation climb. Namely, the direction of the osmotic force is vertical to the slip plane, i.e., parallel to $K^{\left(j\right)}$. To describe the NEVC effects, an extra force acting on the lattice defects can be introduced to mimic the osmotic force. Unfortunately, the APFC model cannot handle the lattice defects directly. Thereby, the osmotic force formulated based on the classical dislocation theory is inapplicable to the APFC model. To solve this problem, we reinterpret the APFC system as the one consisting of the local lattice elements defined in each mesh node. For a given lattice structure, the state of the local lattice element is uniquely defined by the complex amplitudes and the average density. The latter ones evolve according to Equations (14) and (15). Note that all potential rotations of the local lattice elements are automatically incorporated in the complex amplitudes. This facilitates our analyses below. For solids, the average density evolution is not important and has been omitted in many APFC models, for example, the ones in the studies [20,21,32], while the complex amplitude evolution governs the motion of lattice defects in crystals. It is known that Equation (14) only describes the slow diffusion process. To incorporate the contributions of the osmotic force into the complex amplitude evolution, an extra term can be appended to the right hand of Equation (14), i.e.,

(19) $$\frac{\partial \eta_{j}}{\partial t}=-M_{\eta_{j}}\left\{-\left(\epsilon +g\bar{\psi }-\lambda {\bar{\psi }}^{2}\right)\eta_{j}+2\lambda \sum_{k=1}^{N}{\tilde{c}}_{jk}\eta_{j}{\left|\eta_{k}\right|}^{2}+f_{s,\eta_{j}^{*}}^{’}+G_{j}{}^{2}\eta_{j}\right\}+\Lambda_{0}\eta_{j}\varsigma_{p_{j}},\;\left(j=1,2,\ldots ,N\right)$$

where $\Lambda_{0}$ is a model parameter and

(20) $$\varsigma_{p_{j}}=\exp\left(-i\delta \varphi^{\left(j\right)}\right)-1.$$

The argument change $\delta \varphi^{\left(j\right)}$ for each complex amplitude is caused by the potential osmotic force. Because the osmotic force only acts on the lattice defects, $\varsigma_{p_{j}}$ or $\delta \varphi^{\left(j\right)}$ should be zero everywhere other than the position around the lattice defects. This is the basic requirement that must be satisfied by $\delta \varphi^{\left(j\right)}$. It is known that the APFC model always evolves the system towards the minimum-energy state. The presence of the extra term in Equation (19) acts as a determinative noise which gives the system a certain possibility of deviating from the minimum-energy path during the evolutions. In this way, the “rare events” associated with the interactions between the vacancies and the lattice defects, typically not energetically preferred, can be incorporated into the APFC model. This makes the APFC model behave like a Monte Carlo method but operate at the continuum level. Below, we provide an approach to determine $\delta \varphi^{\left(j\right)}$ and thus $\varsigma_{p_{j}}$. For presentation convenience, we refer to such a model as the phase-relaxed APFC (PAPFC) model. 

Assuming that the local lattice element is characterized by $\bar{\psi }$, $\left\{\eta_{j}\right\}$ and $\left\{K^{\left(j\right)}\right\}$, the crystal has an attempt to relieve the local elastic stresses through adjusting strains or $K^{\left(j\right)}$ of the local lattice elements. The minimal model to describe the relaxation of $K^{\left(j\right)}$ within a virtual time interval (δt) is

(21) $$\frac{\delta K^{\left(j\right)}}{\delta t}=-M_{K^{\left(j\right)}}\frac{\delta {ℱ}^{cg}}{\delta K^{\left(j\right)}},\;\left(j=1,2,\ldots ,N\right)$$

where $K_{\alpha }^{\left(j\right)}$ is the α-th component of $K^{\left(j\right)}$ and $M_{K^{\left(j\right)}}$ is the corresponding mobility parameter. Because of the symmetry of $K^{\left(j\right)}$ (*j* = 1, 2, 3), we take $M_{K^{\left(j\right)}}$ to be $M_{K}$ for all $K^{\left(j\right)}$ (*j* = 1, 2, 3) in the present work. By inserting Equation (11) into Equation (21), performing the variational derivative, and rearranging the results terms according to the derivatives of $\eta_{j}$, we obtain the following compact form:

(22) $$\delta K^{\left(j\right)}/\delta t=-2M_{K}\nabla \cdot K^{\left(j\right)}\nabla {\left|\eta_{j}\right|}^{2}+M_{K}{\tilde{p}}^{\left(j\right)},\;\to \left(j=1,2,\ldots ,N\right)$$

where

(23) $${\tilde{p}}^{\left(j\right)}=\left(\eta_{j}^{*}G_{j}\eta_{j}+\eta_{j}G_{j}{}^{*}\eta_{j}^{*}\right)K^{\left(j\right)}-i\eta_{j}^{*}\left[\nabla G_{j}\eta_{j}+\left(1-K_{j}^{2}\right)\nabla \eta_{j}\right]+c.c.,$$

Previously, we proved that ${\tilde{p}}^{\left(j\right)}$ relates to the Cauchy stress (σ) by reference [25]

(24) $$\sigma =f^{cg}I+\sum_{j}{\tilde{p}}^{\left(j\right)}⊗K^{\left(j\right)},$$

where $f^{cg}$ is the coarse-grained free energy density and $\delta_{\alpha \beta }$ is the Kronecker delta. To the zero-th approximation, Equation (22) reduces to

(25) $$\delta K^{\left(j\right)}/\delta t=M_{K}{\tilde{p}}^{\left(j\right)},\;\left(j=1,2,\ldots ,N\right)$$

Note that the first term is small compared with the second term in Equation (24) when the initial pressure is zero. Then, it is easy to find that the variation of $K^{\left(j\right)}$ given by Equation (25) is caused by the elastic relaxation due to the force acting on the local lattice element along $K^{\left(j\right)}$, i.e., a climb force ${\tilde{p}}^{\left(j\right)}$. Under the elastic equilibrium state, such climb force should be equal to the osmotic force. Thereby, Equation (25) naturally describes the influence of the osmotic force associated with the slip plane normal to $K^{\left(j\right)}$. 

Next, we should convert the virtual variation of $K^{\left(j\right)}$ into the argument change. Supposing that the extra displacement due to the relaxation of $K^{\left(j\right)}$ is denoted by ${\tilde{u}}^{\left(j\right)}$, the argument change in $\eta_{j}$ can be expressed as

(26) δφ(j)=K0(j)·u˜(j), (j=1,2,…,N)

where K0(j) represents the *j*-th unstrained RLV and relates to the deformed one by K(j)=K0(j)+δK(j). The compatibility condition of the deformed local lattice elements requires

(27) K(j)·a(j)=K0(j)·(a(j)+u˜(j)) or K0(j)·u˜(j)=K(j)·a(j)−2π. 

where $a^{\left(j\right)}$ is the ideal lattice vector satisfying K0(j)·a(j)=2π. With Equations (25) and (27), Equation (26) can be rewritten as

(28) $$\delta \varphi^{\left(j\right)}=M_{K}\delta t{\tilde{p}}^{\left(j\right)}\cdot a^{\left(j\right)}$$

According to Equation (23), it is not hard to find that ${\tilde{p}}^{\left(j\right)}$ is zero everywhere other than the positions around the lattice defects. This meets the requirement for $\delta \varphi^{\left(j\right)}$ mentioned at the beginning of Section 2.3. If not mentioned, δt coincides with the timestep used in the simulations. When the lattice vector is taken to be a(j)=2πK0(j)/‖K0(j)‖2, Equation (28) can be further rewritten as

(29) $$\delta \varphi^{\left(j\right)}=2\pi M_{K^{\left(j\right)}}\delta tp^{\left(j\right)},\;\left(j=1,\ldots ,N\right)$$

where $p^{\left(j\right)}$ is given by

(30) p(j)=p˜(j)·K0(j).

With Equations (23), (29) and (30), $\delta \varphi^{\left(j\right)}$ can be determined at each timestep after the complex amplitudes are solved. 

Before ending this part, it is necessary to emphasize that the modified APFC model proposed by us has been extensively examined in crystals under various NEVCs in our recent work [25]. It is found that the modified APFC model can describe the climb behaviors of dislocations under different NEVCs. Particularly, $M_{K^{\left(j\right)}}$ can be interpreted as a measure of the relative NEVC. Although the original idea of such a model is inspired by the influence of vacancies on the edge dislocations in the 2D systems, the resulting model exhibits a strong prediction ability for microstructure evolutions at the atom level, not only in the 2D crystals but also in the 3D crystals. For example, such a model can naturally predict various point defect-mediated behaviors of dislocation loops in radiated BCC crystals known in experiments, such as the shrinking, 1D diffusive motion and changing habit plane of dislocation loops. In this work, we will further apply such a model to explore the role of the NEVC effects during the crystallization under deep supercooling. In fact, the determinative noise is determined by the system temperature implicitly. For example, near the equilibrium melting point, the elastic stiffness of the solid approaches zero, corresponding to a very small equilibrium amplitude. According to the idea of our model (See Equations (23) and (29)), it can be inferred that the extra term introduced is also very small. When the system is under supercooling, the elastic stiffness of the solid is large, corresponding to a large equilibrium amplitude. Then, the extra term is also large. The character enables us the explore the solidification behaviors under various supercooling in a natural way.

## 3. Nonequilibrium Solidifications of Hexagonal Crystals

### 3.1. Method and Simulations

Numerical simulations are performed by solving the motion equations for the complex amplitudes as well as that for the average density, i.e., Equation (15). Note that detailed motion equations for the complex amplitudes depend on the detailed model, as described separately in Section 2. A finite element code with adaptive mesh techniques is implemented for the model with a C++ library deal.II [33]. Particularly, the extra term introduced in the PAPFC model is evaluated using a special explicit algorithm [25] during the time integration of the complex amplitudes. Below, we will adopt the PAPFC model to explore the nonequilibrium crystallization behaviors and compare the results with that of the hyperbolic APFC model where necessary. 

**Figure 1:**
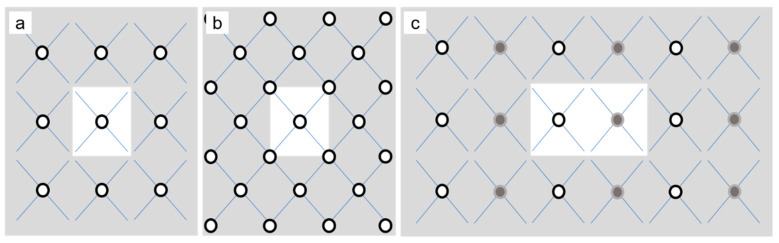
Initial sites of crystallites in (**a**) sample II, (**b**) III and (**c**) IV, where the white region is the actual domain simulated because of the periodic boundary conditions. The white (gray) circle denotes seed with *θ* = 0° (15°), and the blue grid shows the background lattice symmetry, i.e., hexagon (Only two edges are shown for concision). Note that the distribution of samples I and IV are the same except for the seed types. Overall, the seeds in samples II and IV are rectangle-distributed (due to the constraints of the lattice symmetry and the periodic boundary conditions), while the seeds in sample III are hexagon-distributed.

Considering the thermodynamic equilibrium condition of $\epsilon =37{\bar{\psi }}^{2}/15$, we fix the initial $\bar{\psi }$ at −0.2 and explore different ϵ changing within the range of [0.1, 0.2] to check the role the undercooling played on the crystal growth of the hexagonal phase. Four hexagonal samples with periodic conditions applied along X and Y directions are adopted to investigate the effects of seed distribution and its initial rotation angle (*θ*). To this end, nine seeds with different rotation angles uniformly distribute over the first sample (I), the center of the second sample (II) is placed with a seed of *θ =* 0°, the center as well as the four corners of the third sample (III) is placed with a seed of *θ =* 0°, and the positions of (L_x_/4, L_y_/2) and (3L_x_/4, L_y_/2) in the fourth sample (IV) are placed with two seeds whose *θ* is 0° and 15°, respectively. L_x_ (L_y_) represents the size of the corresponding sample along the X (Y) direction. In our simulations, the seeds are generated by placing a small circular grain with a radius of about 1.5*a*_hex_ and the corresponding rotation angle at the aforementioned positions for each sample. It is found that the seeds may not sustain their original states during the simulations without additional constraints. In this case, the seeds of different rotation angles, in essence, provide nucleation sites with different initial states. This can mimic heterogeneous nucleation processes of pure liquid phase with various potential nucleation sites. We mainly focus on such a case, while the results of the seeds with a fixed radius are provided in the Supplementary Materials. We will adopt this scheme to generate the seeds in the four samples. The influences of the seed spacing are examined by adjusting the dimension (*L_x_* × *L_y_*) of the samples. For the first three samples, the explored dimensions range from 1024*a*_hex_ × 512√3*a*_hex_ to 4096*a*_hex_ × 2048√3*a*_hex_. The dimension of sample IV is 4096*a*_hex_ × 1024√3*a*_hex,_ where the seed distribution is comparable to the one of sample II (See Figure 1). The lattice parameter *a*_hex_ is 4*π*/√3 in a dimensionless unit. Without losing generality, initial unrotated amplitudes are assumed to be real and equal in magnitude, whose value is obtained to be $\eta_{0}=\left(-3\bar{\psi }\pm \sqrt{15\epsilon -36{\bar{\psi }}^{2}}\right)/15$ through minimizing the free energy. The minimal grid size is *a*_hex_, and the timestep (Δt) ranges from 0.1 to 0.25 in the dimensionless unit, depending on the value of ϵ. $\Lambda_{0}$ has a dimension of the time inverse and is taken to be 1/Δt. $M_{K^{\left(j\right)}}$ is taken to be 1.0 × 10^−3^. Larger $M_{K^{\left(j\right)}}$ will make the GFN take place in advance and thus affect the atomic structures of the growing crystal. This is because $M_{K^{\left(j\right)}}$ controls the amplitude of the determinative noise (a kind of short-wave interaction), which is crucial for the formation of the spherulites. However, the solidification pattern is qualitatively the same (See Supplementary Materials). 

### 3.2. Crystallization of the Polycrystal under Deep Supercooling

**Figure 2:**
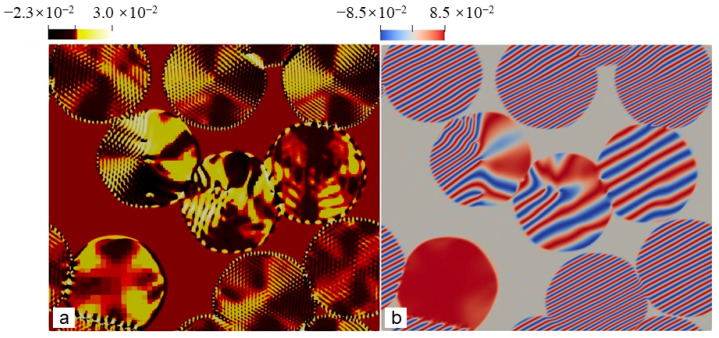
(**a**) and (**b**) are spherulites crystallized at t = 1800 in the sample I with ϵ=0.125. The field plotted in (**a**) are shear stress, defined by $\left(\sigma_{11}-\sigma_{22}\right)/2$ where $\sigma_{ij}$ (*i*, *j* = 1, 2) is the stress component. The stress is calculated using Equation (24), while the field plotted in the figure (**b**) is Re(η1).

Figure 2 shows the polycrystal growth morphology in sample I at relatively moderate undercooling, but still deep supercooling. The distortion degree of the local lattice in the growing crystals can be observed from the shear stress field (See Figure 2a). It is known that each spherulite contains multiple subgrains with different orientations [3]. The lattice distortion near the boundary between the adjacent subgrains should be distinctly different from the subgrain interior. As shown in Figure 2a, the part of a grain with a relatively uniform color represents a subgrain. Because the orientation of each subgrain within a grain is different, the stripe spacing in the Re(η1) field should be different for these subgrains due to the different rotations, which agrees with the results shown in Figure 2b. The formation of the spherulite arises from incoherent local lattice rotations caused by the NEVC effects during the crystallization. Depending on the relative rotation angle between adjacent grains separated by a grain boundary, the separation distance between adjacent boundary dislocations may be larger than the grain size so that the boundary dislocations may not be observed in our results because of the limited grain size, for example, in the upper three grains in Figure 2a,b. In other cases, boundary dislocations emerge in the grain interior. Thereby, it can be concluded that the growing grains in Figure 2b or Figure 2c are spherulites, which are extremely similar to experimental ones [3].

**Figure 3:**
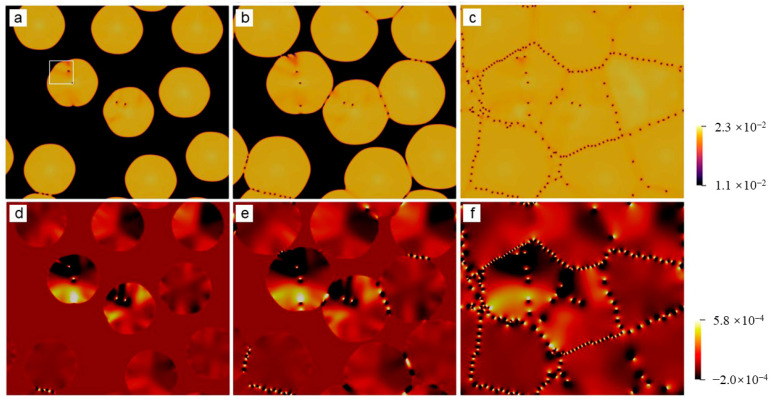
Nonequilibrium crystallization process of the polycrystal (sample I with the size of 512*a*_hex_ × 256√3*a*_hex_) was simulated using the PAPFC with ϵ = 0.125. The field plotted is (**a**–**c**) A^2^ or (**d**–**f**) P, where each field is shown at three moments (i.e., t = 1400, 1800 and 3320 in dimensionless unit) sequentially from left to right.

The whole formation process of the polycrystal under moderate undercooling is shown in Figure 3. Morphologies of the growing grains and dislocations in the grains are visualized using A^2,^ which is nearly zero at the dislocation cores, is precisely zero in the liquid and is nonzero in crystals. The dislocation, as well as the Burgers vector, can be identified by the pressure ($P=-\sum_{j=1}^{N}p^{\left(j\right)}/d$, *d* is the dimension of the system) field in the growing grains (See Figure 3d–f). Each dislocation core is surrounded by a pair of pressure extremes, i.e., the local maximum (denoted by bright yellow) and minimum value (denoted by black), in the pressure field. The Burgers vector of the dislocation is vertical to the direction pointing from the black side to the yellow side. It will be further examined in the next paragraph. The results shown in Figure 3 indicate that the dislocations can form in the interior of the grains during grain growth. From Figure 3f, it is found that the Burgers vectors of the dislocations belonging to the same straight grain boundary are the same. This means that a row of dislocations with the same Burgers vector actually forms a grain boundary. Thereby, the dislocations in the grain interior are, in fact, the grain boundary dislocations that divide a grain into several subgrains, for example, the grain with a white square in Figure 3a. This further confirms that the growing grains are actually spherulites. 

**Figure 4:**
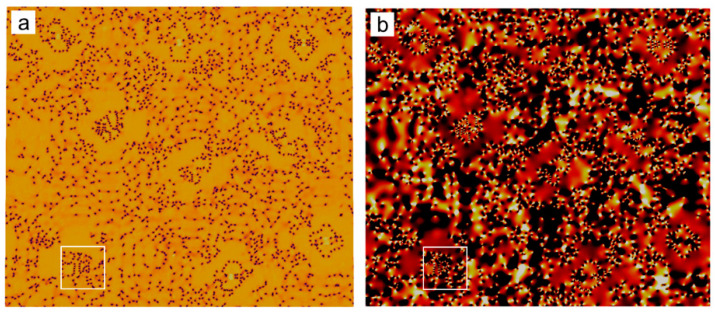
The field plotted is (**a**) A^2^ or (**b**) P of the polycrystal simulated using the PAPFC model. The undercooling parameter is ϵ=0.2. The color schemes in (**a**) and (**b**) are the same as the ones in Figure 3a–c,d–f, respectively. The snapshot corresponds to the time of 495 in dimensionless unit. The region marked by the white square in (**a**) is the same as that in (**b**).

**Figure 5:**
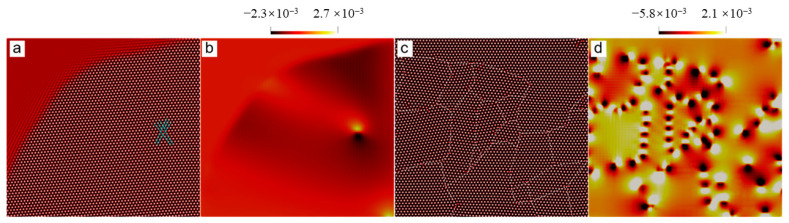
Reconstructed atom density of the region marked by the white square in (**a**) Figure 3d and (**c**) Figure 4a; (**b**) and (**d**) are the pressure field of the same region as (**a**) and (**c**), respectively. In (**a**), a dislocation configuration is depicted by the cyan lines. The white lines in (**c**) correspond to the position of grain boundaries.

At the elevated undercooling, patterns of the growing grains become extremely complex, which depends on several factors, such as seed spacing and seed distribution, among others. More details will be discussed in details later in Section 3.3. Below, we focus our attention on the microstructures of the polycrystal formed under high undercooling. Figure 4 shows the final crystallization morphology of sample I with ϵ=0.2 after the solidification finishes. The dislocation distribution can be identified using the field of A^2^ or the pressure. The result shown in Figure 4 suggests that the dislocation density is extremely high, and the grains are so small that they are nearly indistinguishable. It can be inferred that an amorphous phase will be formed when the undercooling (and thus the dislocation density) is sufficiently high (large). This obeys present understandings of the nonequilibrium solidifications. The core structures of dislocations are examined from the atom density field reconstructed using Equation (6). Figure 5 shows the reconstructed atom density field for the case of ϵ = 0.125 and 0.2. From Figure 5a,b, it is found that the dislocation core can be well identified from the pressure field, and the dislocation, in fact, consists of two edge components whose half-planes of atoms are depicted with the cyan lines. This result indicates the resulting Burgers vector is indeed vertical to the direction pointing from the local minimum to the local maximum pressure. The results shown in Figure 5c,d indicate that the crystallization under the high undercooling result in quantities of small grains.

### 3.3. Nonequilibrium Patterns during the Growth of a Single Seed in the Presence of Other Potential Seeds

**Figure 6:**
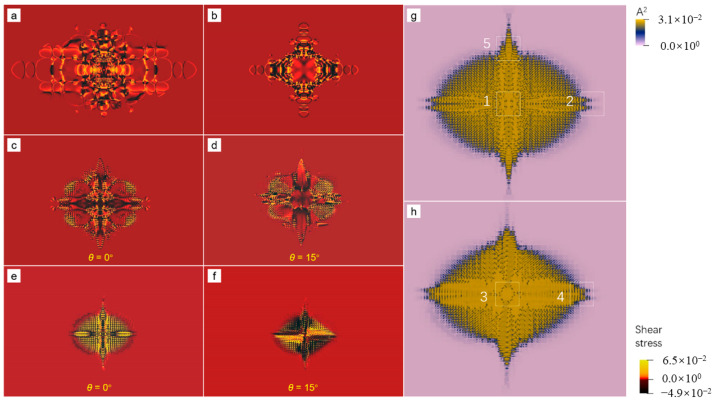
Morphology of crystal growth in the different samples with various undercoolings. (**a**) is columnar dendrite in sample II with ϵ=0.125. (**b**) is equiaxial dendrite in sample III with ϵ=0.125. (**c**,**d**) are equiaxial dendrites in sample IV with ϵ=0.125 and (**e**,**f**) are the same but with ϵ=0.15. The field plotted in (**a**–**f**) is the shear stress. Lattice defects (mainly dislocations) in (**e**) and (**f**) can be observed more clearly from the A^2^ field plots, i.e., (**g**) and (**h**), where five regions, marked by the white squares, have a size of 500 × 500 for each.

To make the pattern formation mechanism clear, we resort to the growth kinetics of a “single” seed. Actually, the seed is still in a polycrystal but with much larger seed spacing because of the periodic boundary conditions. The results are shown in Figure 6. In particular, Figure 6a–f are visualized using the corresponding shear stress field, which can not only distinguish the shape of the growing grain (having nonzero shear stress), but also highlight the subgrain boundaries (having a larger positive or negative value corresponding to the bright yellow or black color in the figures). Surprisingly, at moderate undercooling (ϵ=0.125), a microscopic columnar-to-equiaxial transition takes place when the seed distribution changes from rectangle symmetry (Figure 6a) to hexagonal symmetry (Figure 6b). This can be attributed to the combined effects of the long-wave and the short-wave interactions. The long-wave effect described by the APFC model result in the crystalline state invading deeply into the metastable liquid under the supercooling [24] so that the adjacent growing grains or growth fronts interact with each other before they actually impinge. The effective interaction distance grows with the increment of the undercooling and can well cover the seed spacing explored in this work. As a result, the crystallization patterns are sensitive to the initial positions of the seeds. Our result indicates that the interaction between different seeds slows down the growth speed. Thereby, it can be inferred that the pattern spreads more slowly along the direction along which the seed spacing is smaller, which explains the microscopic columnar-to-equiaxial transition. The role of the short-wave interaction, i.e., the determinative noise introduced by the PAPFC model, is critical for the GFN, which is the major growth mechanism under deep supercooling. They will be further discussed in Section 3.4 and Section 3.5. Below, we focus on the characteristics of the nonequilibrium patterns during the crystallization. The columnar dendrite is prone to growing along the X axis of sample I due to the relatively large seed spacing, while the equiaxial dendrite grows simultaneously along the X and Y axes of sample II. Keeping the rectangular symmetry but rotating the right (as well as the left) seed by 15°, the columnar dendrite becomes more of a nonsymmetric equiaxial dendrite with distinctly different primary arms. Except for the four primary arms growing along X and Y directions (See Figure 6c,d), multiple side arms are observed in the nonsymmetric equiaxial dendrite. The influence of the initial rotation angle of the seed on the pattern is due to the anisotropic surface energy, which affects the major growth direction at the early stage. Consequently, the interaction between adjacent grains happening at the later stage is modified, which eventually influences the crystallization pattern. Further increasing the undercooling, the dendrite turns out to be even slenderer (See Figure 6e,f). Interestingly, the growth tip of the left (or right) primary arm is split (See Figure 6c,d,g,h), resembling the tip splitting of dendrites arising from anisotropic interface energy [34,35]. It is found that the tip splitting is formed through the GFNs. In the present work, such a phenomenon can be explained by the interactions between the invading crystalline states among the major growth fronts, which create the preferred nucleation sites for the GFNs with the aid of determinative noise. Dislocation density is much larger in regions between the primary arms (See Figure 6g,h) due to the impinging of small grains formed by the frequent occurrence of the GFN. This coincides with that of the polycrystal under high undercooling. The envelope of the nonsymmetric equiaxial dendrite is rectangular (or ellipsoid). Considering the rectangle-distributed seeds (See Figure 1), such a result can be well interpreted by the aforementioned long-wave interactions.

**Figure 7:**
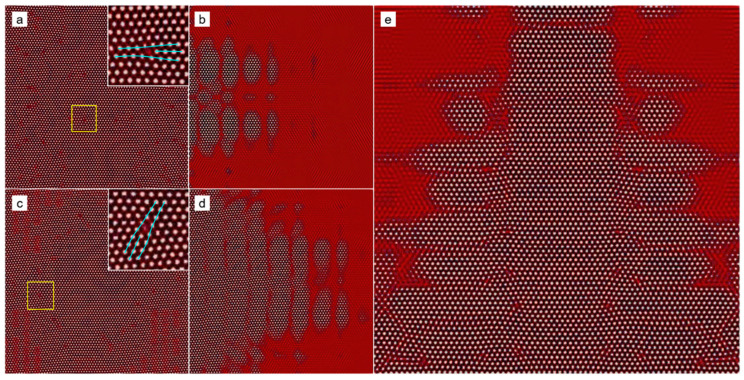
Reconstructed atom density field of the five regions in Figure 6g,h. (**a**–**e**) correspond to the region numbered from “1” to “5”, respectively. The inset in (**a**) or (**c**) is the magnified view of the corresponding region marked by the yellow square, where the core structures of the dislocations are clearly seen.

Typical local microstructures of the nonsymmetric equiaxial dendrites at the atom level are reconstructed, and the results are shown in Figure 7. Dislocation configurations at the atom level can be clearly identified from the results (Figure 7a,c). Interestingly, dislocations mainly emerge outside a circular region centered at the seed (such as the region “1” and “3” in Figure 6g,h). After a sufficient relaxation time, these dislocations would partly annihilate, accompanied by rotation and shrinking of the small grains. The relaxation rate relies on the competition between the diffusion and elastic relaxation processes, which exceeds the scope of the present work. In addition, the GFN at the growth tip and the dendric growth with side arms are observed (See Figure 7b,d,e). The reason for such behaviors is, in essence, the same as that of the microscopic columnar-to-equiaxial transition, i.e., the invading crystalline states in the liquid phase. In this case, the invading crystalline states mainly come from the different growth fronts of the same grain. The reason is as follows. There are several major growth fronts for a grain, which is initially because of the anisotropic surface energy. The invading crystalline states generated by the adjacent major growth fronts interact with each other. As a result, the determinative noise due to the NEVC effects is much stronger in the liquid where the interaction begins, which stimulates the nucleation of new crystallites with different orientations. The mechanism for such a process will be further discussed in the next part.

According above discussions, we reproduced many typical nonequilibrium patterns, including spherulites, columnar dendrites and equiaxial dendrites, during crystal growth by the PAPFC model. Moreover, our results uncover a microscopic columnar-to-equiaxial transition, which was not well understood previously, depending on the undercooling, lattice symmetry and seed distributions. Dislocation density notably increases only after a certain period of crystal growth and becomes larger with the promotion of undercooling. 

### 3.4. The Crystal Growth under the Deep Supercooling

**Figure 8:**
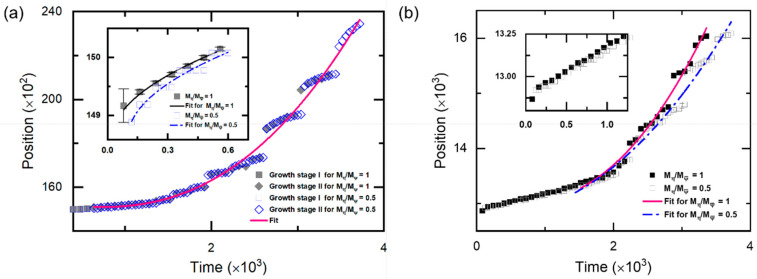
(**a**) Right-growth front position versus time for sample II with ϵ=0.125, where the gray solid line (or blue dot–dash line) for growth stage I in the inset is a fit to relation [36]: $Z=Z_{0}+C\sqrt{t-t_{0}}$, $Z_{0}=14858.64$, $t_{0}=20.00$ and C=6.59 (or $Z_{0}=14861.66$, $t_{0}=102.06$ and C=6.60), and the pink solid line for growth stage II ($M_{\eta }/M_{\bar{\psi }}=0.5$) is a fit to $Z=Z_{0}+C{\left(t-t_{0}\right)}^{n}+Ae^{-\left(t-t_{0}\right)/\tau_{0}}$, $Z_{0}=14262.90$, $C=5.15\times 10^{-5}$, n=2.36, $t_{0}=638.72$, A=828.90 and $\tau_{0}=2.92\times 10^{3}$. (**b**) Upper-growth front position versus time for the sample II with ϵ=0.125, where the pink solid line (or blue dot–dash line) is a fit to the same relation satisfied by stage II for the case of $M_{\eta }/M_{\bar{\psi }}=1$ (or $M_{\eta }/M_{\bar{\psi }}=0.5$), and the fitting parameters are $Z_{0}=12997.78$, $C=3.78\times 10^{-5}$, n=2.30, $t_{0}=539.89$, A=0.16 and $\tau_{0}=1.70\times 10^{2}$ (or $Z_{0}=12747.83$, $C=6.58\times 10^{-5}$, n=2.17, $t_{0}=11.37$, A=4.88 and $\tau_{0}=1.41\times 10^{2}$). The inset is the magnified view of the first growth stage.

**Figure 9:**
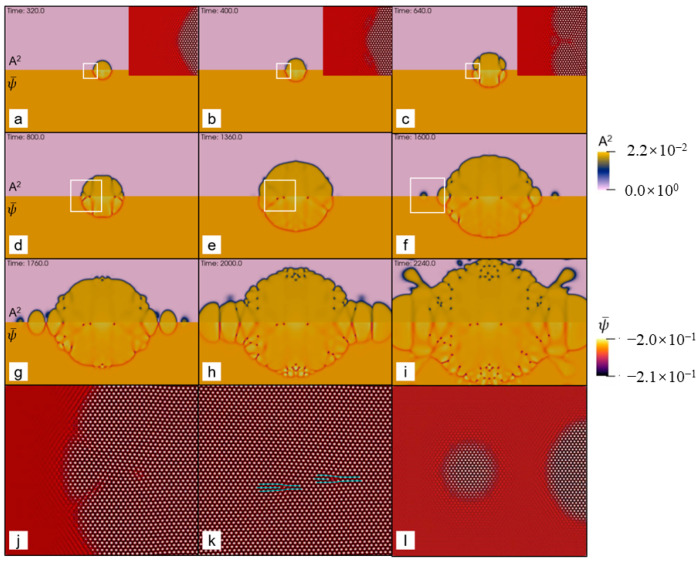
Enlarged contour plots at different moments showing the columnar growth processes of the hexagonal phase at ϵ=0.125. In (**a**–**i**), the upper panel plotted is field A^2,^ while the lower is $\bar{\psi }$. Reconstructed atom density fields of the region marked by the white rectangles in (**a**–**f**) are given in the inset of (**a**–**c**) and in (**j**–**l**) for (**d**–**f**), respectively. Although initial simulated system is centrosymmetric, dislocations generated during the crystal growth slightly deviate from the centrosymmetric distribution. This is because the initial seed may not be strictly centrosymmetric after the numerical discretization.

The kinetics of the right (upper) growth front for sample II is shown in Figure 8a,b. Two growth stages can be identified. In the first growth stage, corresponding to growth stage I in Figure 8a, the crystal grows mainly through diffusive transport of mass, i.e., slow mode, whose interface velocity (*v*) vs. growth time (*τ = t* − *t*_0_, *t*_0_ is the nucleation time) characterized by the relation of $v\propto \tau^{-1/2}$. This result agrees with the one [36] predicted by the diffusive PFC model, except for a larger velocity coefficient because of the higher undercooling. The density of lattice defects characterized mainly by dislocations is small in this stage. As a result, a relatively “clean” region at the center of the growing crystal is observed (See Figure 9a,b). With the growth of the clean region, the local low-density layer appears at the growth front due to the increasing depletion layer. The emergence of the low-density layer arises from the increment of the average density in the solid at the “clean” region, where the color representing the density is slightly brighter than the liquid in Figure 9. In contrast to the solidifications near the thermodynamic equilibrium state, the following growth can proceed in the unstable liquid without apparently increasing its density. This can be found in Figure 9d–i, where the color representing the density within the growth front is almost the same as that in the liquid. This is reasonable because the liquid under deep supercooling is less energetically stable than the corresponding solid phase, even with the same density. As the crystal continues to grow, the higher density region spreads from the center to its surroundings, but at a speed much slower than that of the crystal growth. In addition, the interactions between the invading crystalline states generated by the major adjacent growth fronts can be identified from the inset of Figure 9b,c. The GFN is prone to occur in the interaction region. The determinative noises due to the NEVC effects are of key importance for the occurrence of the GFN with a different orientation. The newly formed grain seed grow and, in turn, generate the new invading crystalline state interacting with the old ones. The new interaction promotes the next GFN. Such a process repeats again and again during the second growth stage. This accounts for the GFN-dominated growth processes, i.e., growth stage II shown in Figure 8a. A notable feature in this stage is that the growth front moves forward through a combined mechanism of diffusion-controlled anisotropic growth and GFN-controlled growth, which leads to the step-growth style of the front (See Figure 8a). Within each “step”, the crystal grows still through the diffusion-controlled mechanism. In contrast to the steady growth speed observed in solid–liquid coexistence regime from both PFC simulations [11] and experiments on colloidal hard sphere crystallization [37], the interface velocity vs. growth time asymptotically approaches to a relation of $v\propto \tau^{n-1}$ at sufficient growth time, where n~2 in the present work (See Figure 8). We further investigate the crystal growth in sample II under different undercooling. The two stages are also observed at the lower or higher undercooling (See Supplementary Materials). 

**Figure 10:**
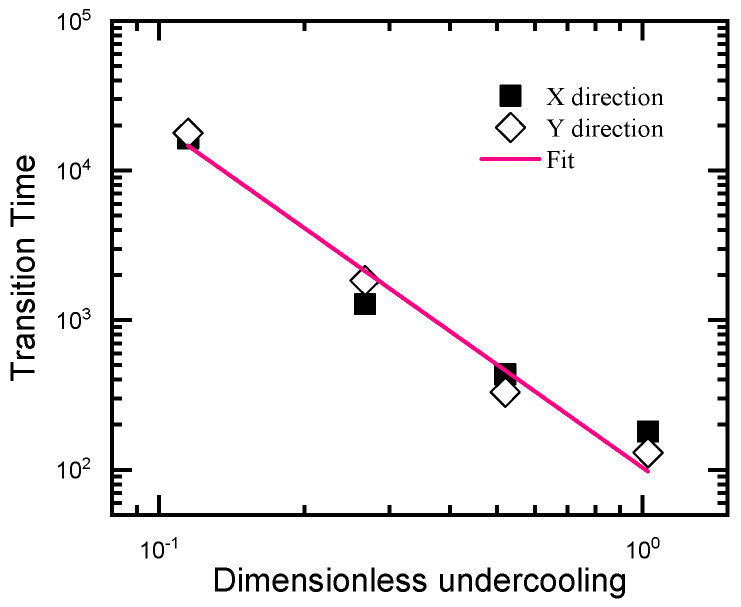
Transition time between growth stages I and II versus undercooling reduced by the equilibrium ϵ. The pink solid line is a fit to a power law, satisfying $t_{I↔II}=103.58*{\left(\epsilon /\epsilon_{0}\right)}^{-2.29}$.

In particular, to examine the role of the ratio of $M_{\eta }/M_{\bar{\psi }}$ on the crystal growth, we modify the ratio of $M_{\eta }/M_{\bar{\psi }}$ by setting $M_{\bar{\psi }}=2.0$. Results for the kinetics of the right (upper) growth front are shown in Figure 8. The two growth stages also emerge, but are slightly different quantitatively. Larger diffusion mobility of the average density slows down the growth speed along the X direction in the first stage as well as the growth along the Y direction in the second stage, while it has a tiny influence on the growth along the X direction in the second stage and that along the Y direction in the first stage. The smaller growth speed caused by the large diffusion mobility of the average density is due to the comparable mass transportation speed with that of the crystal growth (such as the growth along X during the first stage). As a result, a relatively large low-density region appears in the front of the solid–liquid interface. According to the PFC phase diagram, the undercooling of the low-density region is relatively low compared with that of a smaller $M_{\bar{\psi }}$, as well as the initial undercooling of the system. Then, the growth speed is slower than the later ones. However, if the growth speed of the crystal is much faster than that of the mass transportation (Such as the growth along X during the second stage) or much slower than the latter (such as the growth along Y during the first stage), the influence of $M_{\bar{\psi }}$ is tiny (as observed in Figure 8a). The influence of $M_{\bar{\psi }}$ on the growth along the Y direction at the second growth stage is caused by the interactions of the density field from the adjacent seeds, since the seed spacing along this dimension is relatively small. Moreover, the influence is not obvious at the beginning of the second stage (See Figure 8b) when the adjacent seeds still separate far enough from each other. This further supports the above assertion. The transition time between the two growth stages relates to the undercooling by a power law (See Figure 10). Such a result suggests that the second growth stage emerges in the crystallization system with either large seed spacing and low undercooling or smaller seed spacing and high undercooling. By controlling the transition time in terms of adjusting the undercooling and the seed spacing (related to the system size and impurity content, among others), polycrystals with different grain sizes, grain shapes and dislocation densities can be acquired. 

### 3.5. The Role of the Short-Wave Interaction on the Nonequilibrium Crystallization 

**Figure 11:**
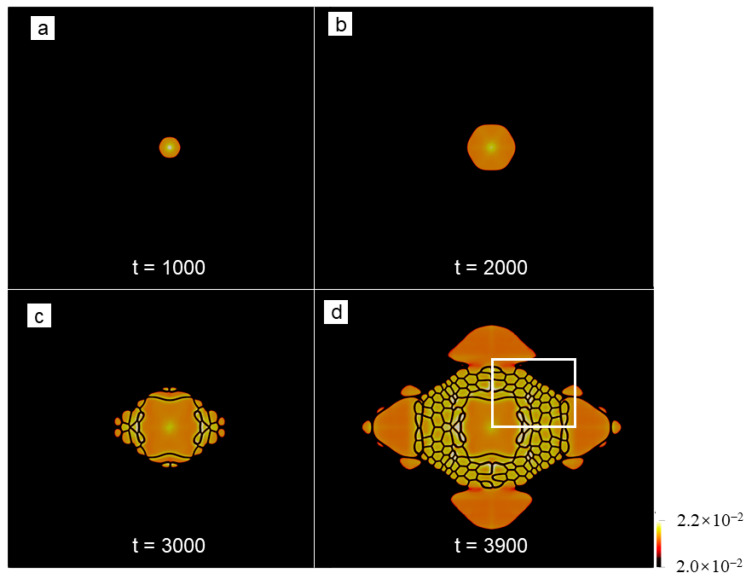
(**a**–**d**) Crystal growth at different times in a relatively small sample II under an undercooling of ϵ=0.125 predicted by the hyperbolic APFC model with τ=1.0. The field plotted is A^2^.

**Figure 12:**
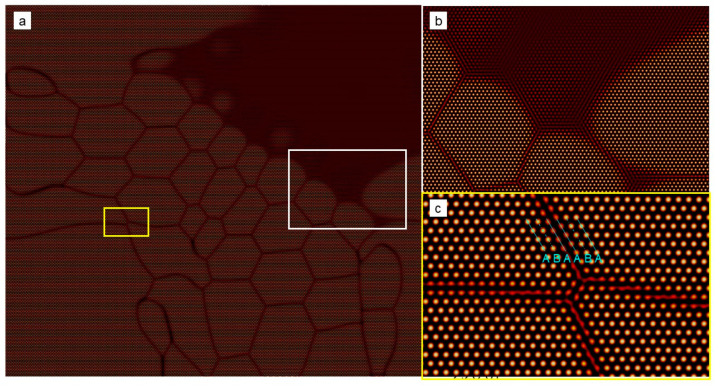
(**a**) Reconstructed atom density field of the region marked by the white rectangle in Figure 11d, where the enlarged view of the region marked by the white or yellow rectangle is shown in (**b**) or (**c**). Particularly, the stacking sequence of the close-packed atom plane is marked out, from which the stacking fault can be identified.

**Figure 13:**
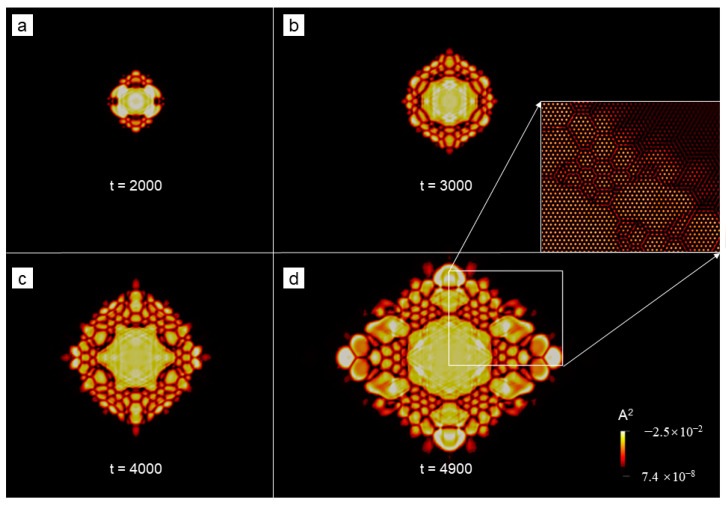
(**a**–**d**) Crystal growth at different times in a relatively small sample II under the undercooling of ϵ=0.125 predicted by the hyperbolic APFC model with τ=1000.0. The inset shows the reconstructed atom density field of the region marked by the white rectangle in (**d**).

**Figure 14:**
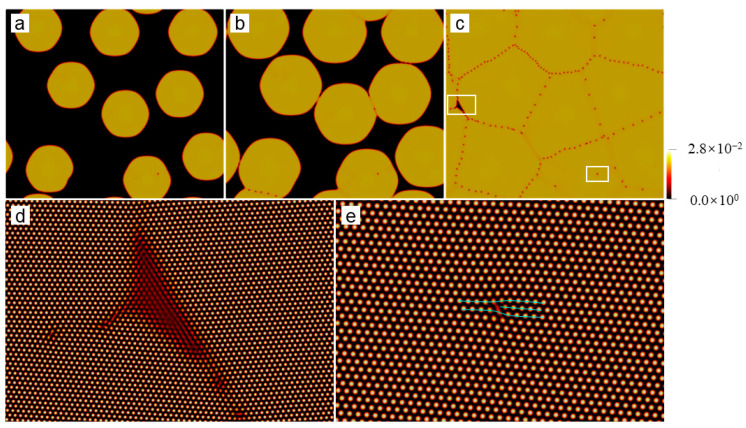
Nonequilibrium crystallization process of the sample I simulated using the hyperbolic APFC model under the undercooling of ϵ = 0.125. The field plotted is A^2^. (**a**–**c**) corresponds to the moments of t = 1400, 1800 and 3300, respectively. (**d**,**e**) are the reconstructed atom density fields of the regions marked by the white rectangle in (**c**). Particularly, (**e**) shows the dislocation in the grain interior, which is generated during the growth.

To examine the role of the short-wave interaction on the crystallization behaviors, we conduct a similar simulation using the hyperbolic APFC model, which contains the short-wave interaction. We select sample II, but with a relatively small size, i.e., 1024*a*_hex_ × 512√3*a*_hex_, as the research object. Considering the influence of the seed spacing or the system size, such a sample is also used for the corresponding PAPFC simulations, and the results are provided in the Supplementary Materials. For the numerical simulations using the hyperbolic APFC model, the minimum grid size is 0.5*a*_hex_, and the timestep is 0.1 in the dimensionless time unit. Two elastic interaction lengths are explored by setting τ to 1.0 and 1000.0. The simulation results for τ=1.0 is shown in Figure 11 and Figure 12. It is found that the envelope of the growing grain is similar to that predicted by the PAPFC model, especially at the early stage (t ≤ 3000), but different in the defect type inside the grain. For example, both of the models predict columnar growth at the early stage. However, at the late stage, when the distance between the growing grains becomes very small, such as the time at t = 3900 in Figure 11d, the growth speed of the GFN increases obviously for the hyperbolic APFC model. Because of the large sample used for the PAPFC simulations, such a stage is not reached within our simulation time. However, this behavior can emerge in the PAPFC simulation for the small sample. The columnar growth is, in essence, arising from the same reason, i.e., the interactions of the invading crystalline state in the unstable liquid ahead of the growth front, which can be well described by the APFC model. However, the GFN cannot occur in the APFC model because of the lack of suitable short-wave elastic interactions. In comparison, the hyperbolic APFC model incorporates a short-wave elastic interaction by introducing the extra inertia term, and the PAPFC model contains the extra determinative noise, which is, in fact, a special kind of short-wave elastic interaction. Different from the dislocations as the major lattice defects present in the PAPFC simulation results, the lattice defects in the growing grain are mainly stacking faults (See Figure 12). With the increment of the interaction length, the growing speed of the grain becomes apparently slower, and the directional growth is less obvious (See Figure 13). In addition, the average grain size is smaller. Considering that large interaction length corresponds to the small critical undercooling [24], i.e., large relative undercooling, this result, in fact, coincides with the result of PAPFC simulations under the high undercooling. Thereby, it could be concluded that, except for the seed spacing and distributions, the elastic interaction length is the key factor that determines the nonequilibrium crystallization patterns as well as the grain size under the different undercooling, while the short-wave elastic interaction plays a key role for the GFN and the major defect types in the final crystals depends on the detailed short-wave elastic interaction type. Interestingly, when multiple grains are present, dislocations may also emerge in the interior of the growing grain for the hyperbolic APFC model (See Figure 14). However, the dislocation density is much lower than that predicted by the PAPFC model. 

## 4. Faceted and Dendritic Growth of BCC Crystals

To further confirm our results obtained from the 2D lattice, large-scale simulations are also conducted to investigate the crystal growth in the BCC lattice using the PAPFC model. A small BCC seed with [100], [010] and [111] aligning along the X, Y and Z axis, respectively, is placed at the center of a simulation box initially filled with an equilibrium liquid phase. The size of the simulation system is 80*a*_bcc_ × 80*a*_bcc_ × 80*a*_bcc_, where the dimensionless lattice parameter *a*_bcc_ is 2√2*π*. Periodic boundary conditions are applied along X and Y directions. Model parameters $\left(\epsilon ,\bar{\psi }\right)$ are selected to be (0.35, −0.35) and (0.40, −0.35), corresponding to low and high undercooling in the phase diagram [38]. Assuming that the initial amplitudes are real and equal in magnitudes (represented by $\eta_{0}$), we obtain $\eta_{0}=\left(-2\bar{\psi }\pm \sqrt{5\epsilon -{\bar{\psi }}^{2}}\right)/15$ after minimizing the free energy functional. The minimal grid size is 1.25*a*_bcc_. The dimensionless timestep is 0.2 for the case of ϵ=0.35 and 0.1 for the case of ϵ=0.40. 

**Figure 15:**
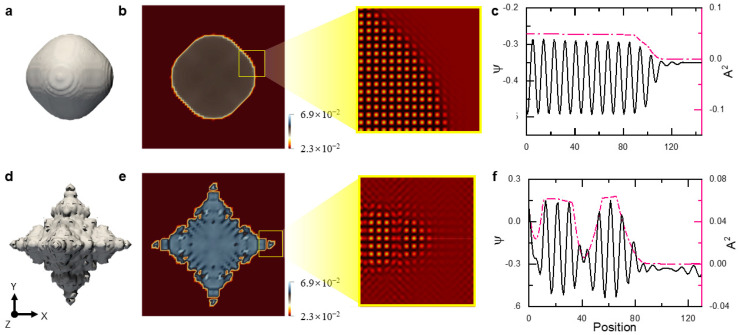
Faceting morphology and equiaxial dendrite in the BCC sample (**a**–**c**) at t = 670 with ϵ=0.35 and (**d**–**f**) at t = 215 with ϵ=0.4, respectively. The contour surfaces in (**a**) and (**d**) correspond to A^2^ = 0.02. The cross section, i.e., Z = 40*a*_bcc_ plane, of the system at the corresponding time is shown to the left of (**b**) for ϵ=0.35 and (**c**) for ϵ=0.4, respectively, where the field plotted is A^2^. To the right of (**b**) or (**e**), the enlarged reconstructed-density view corresponds to the region marked by the yellow square in the left figure, which shows the atomic microstructure of the liquid–solid interface at the right growing tip. (**c**) or (**f**) are the profiles of atom density (*ψ*) and A^2^ along the cyan line drawn in (**b**) or (**e**). It is found in (**c**) that the average density in the liquid phase is larger than that in the solid phase. This is because the cyan line we chose to draw the profile with is not exactly passing through the center of the atoms in the solid phase.

**Figure 16:**
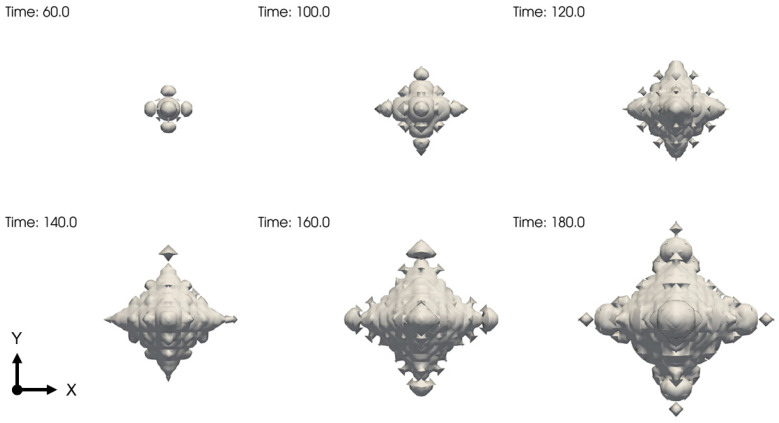
Dendritic growth of the BCC crystal through the GFN mechanism. The contour surfaces are the same as the ones in Figure 15.

The results are shown in Figure 15, where faceting morphology and equiaxial dendrite are observed. The growth fronts for the two cases (corresponding to the two growth stages) are featured by clean and smearing solid–liquid interfaces, respectively (See Figure 15b,c,e,f). Notably, the crystalline state around the smearing solid–liquid interface invades deeply into the liquid. At the low undercooling, the diffusion-controlled growth stage covers the whole simulated crystallization process. When the simulation time (as well as the seed spacing) is sufficiently long (large), the second growth stage emerges, according to the results in Section 3. Alternatively, we can observe such a growth stage by elevating the undercooling with less computational effort. The results at the high undercooling are shown in Figure 15d–f and Figure 16. It is found that the GFN-dominated growth quickly takes over the crystallization process controlled by the mass diffusion and results in formations of the complex nonequilibrium pattern, i.e., the equiaxial dendrite, which confirms our assertions above. In the real crystallization system, the seed spacing is usually much larger than the one explored in the present work. This will lower the undercooling condition required to observe the second growth stage.

## 5. Summary and Conclusions

In summary, nonequilibrium crystallizations are investigated using the PAPFC model and the hyperbolic APFC model. Particularly, the PAPFC model provides a way to explore the solidification behaviors under various supercooling in a natural way due to the implicit dependence of the extra determinative noise on the system temperature. Nonequilibrium patterns, including the faceted growth, spherulite, symmetric and nonsymmetric dendrites, among others at the atom level, are revealed under deep supercooling. Except for the undercooling, it is found that the nonequilibrium pattern during the crystallization depends on the lattice symmetry, undercooling, seed type and interaction among the growing crystallites. Particularly, roles of the interactions arising from seed distributions as well as seed types (characterized by initial rotation angular) are investigated. Our results show that the combined effect of the long-wave and the short-wave elastic interaction is responsible for the pattern formations. The long-wave interactions inhabited in the APFC model result in the crystalline state invading deeply into the metastable liquid under the supercooling so that the growing grains, as well as the growth fronts, interact with each other before directly impinging. At the same time, the short-wave interaction plays a key role in the GFN. Particularly, the major defect types in the final crystals depend on the detailed type of the short-wave elastic interaction. The short-wave interaction due to the inertia effects tends to generate stacking faults in the growing crystals, while the one due to the NEVC effects is prone to generate dislocations. Overall, two growth stages, i.e., diffusion-controlled anisotropic growth and GFN-dominated growth, could be identified. In contrast to the solidification near the thermodynamic equilibrium states, the crystal is found to grow in the unstable liquid without apparently increasing its density at the second growth stage. At the large undercooling, the diffusion-controlled growth stage becomes so short that it is difficult to be aware of its existence compared with the second stage. Dislocations are dramatically generated in the second stage, mainly through impinging of small crystallites formed via the GFN mechanism. This may be the source of the precursor of amorphous nucleation. Nonequilibrium solidifications of BCC crystals are also investigated, which supports the two growth stages. The result of the present work provides clues for designing the polycrystals containing grains with various shapes and sizes, as well as different initial dislocation densities, through controlling the undercooling, seed density, container shape, and so on, to meet the various requirements of application realms. Finally, it should be pointed out that the APFC-based model, either the PAPFC model in this work or the hyperbolic APFC model, could serve as an important atomic simulation method for predicting solidification behaviors under various supercooling, but the typical system size simulated is still limited within about ~1μm at present. Nevertheless, such a method could serve as an ideal bridge between the mesoscopic method (such as the OFPF) and the classical atomic method (such as the classical molecular dynamics).

## Data Availability

The data presented in this study are available on request from the corresponding author.

## Supplementary Materials

The following supporting information can be downloaded at: https://www.mdpi.com/article/10.3390/e25050708/s1, Figure S1: Pattern evolution during the nonequilibrium crystallization of sample II under ϵ = 0.125 using PAPFC with Mk = 1.0 × 10^−3^; Figure S2: Pattern evolution during the nonequilibrium crystallization of sample II under ϵ = 0.125 using PAPFC; Figure S3: Right tip position versus time for sample II with ϵ = 0.1; Figure S4: Dendrite tip position versus time for sample II with different undercoolings; Figure S5: Enlarged contour plots showing the growth processes of the equiaxial dendrite in the main text; Figure S6: Pattern evolution during the nonequilibrium crystallization of sample II under ϵ = 0.125 using PAPFC with Mk = 0.5; Figure S7: Pattern evolution during the nonequilibrium crystallization of the same sample as that in S6 under ϵ = 0.125 using PAPFC with Mk = 2.0 × 10^−3^.
