# Supplementary material for: Pattern Formation under Deep Supercooling by Classical Density Functional-Based Approach
Source: Entropy (Basel). 2023 Apr 24;25(5):708. doi: 10.3390/e25050708 (PMC10217044; doi:10.3390/e25050708)
Supplement: Supplementary file 1 [file entropy-25-00708-s001.zip › entropy-2293948-supplementary.pdf]

Supplementary Materials

As a complement to the main text, we further compare the results simulated with different seed distributions to uncover the influence of the seed distribution on the growth pattern as well as the growth rule (See S1-5). Moreover, the influence of M_k is also checked through comparing the results of M_k = 2.0e-3 and M_k = 0.5 (See S6 and S7).

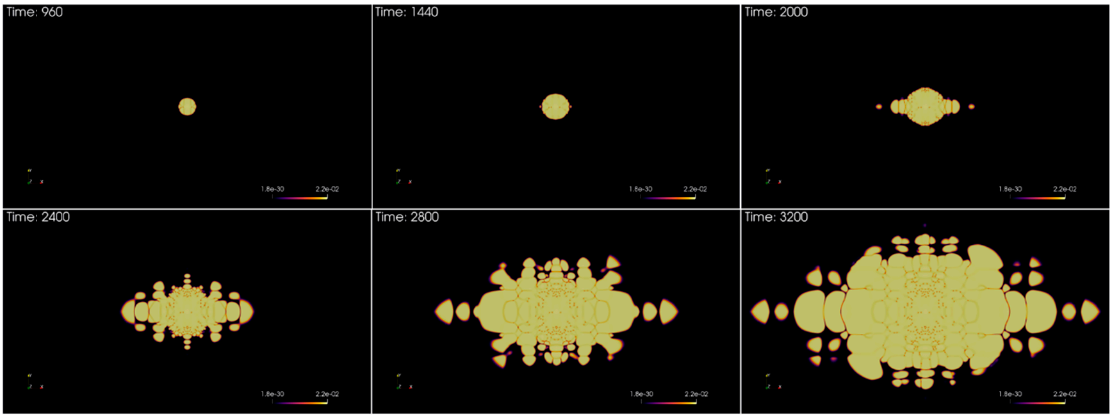

S1. Pattern evolution during the nonequilibrium crystallization of sample II under $\epsilon = 0.125$ using PAPFC with M_k = 1.0e-3. The field plotted is A^2. Other conventions are the same as in the main text.

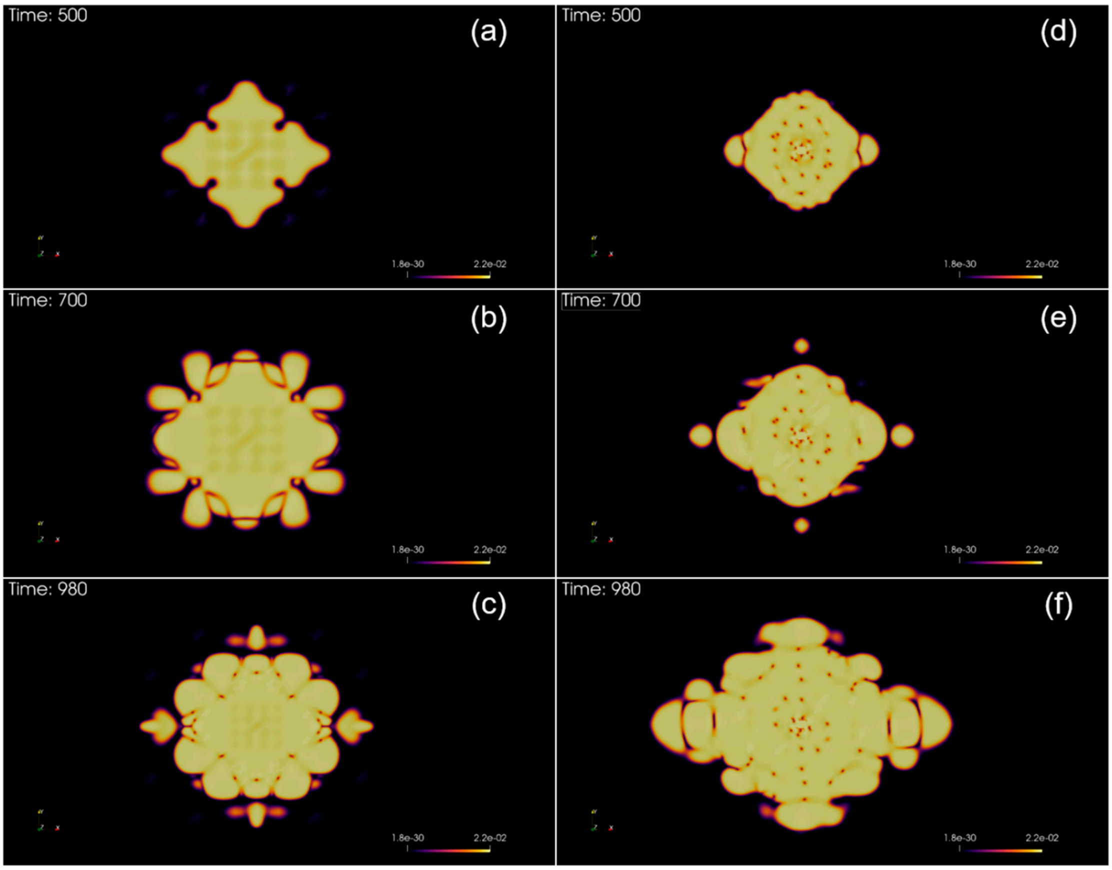

S2. Pattern evolution during the nonequilibrium crystallization of sample II under $\epsilon = 0.125$ using PAPFC, where figure (a-c) shows the patterns of the left grain, and (d-f) shows the ones of the right grain. In contrast to the main text, we fix the radius of the two seeds at about $1.5a_{\mathrm{hex}}$, without relaxations with time. This could simulate the nucleation and growth at impurities in the supercooled liquid. The field plotted is A^2. It can be concluded from the results that the pattern formation also relies on the seed type in addition to the ones mentioned in the main text. Other conventions are the same as in the main text.

Figure S3 shows the kinetics of the right growing tip for sample II at small undercooling, which obeys the same law as the one found by Tegze [1] using the traditional PFC model. The growth is controlled mainly through mass diffusion. However, with the growing undercooling, the diffusion-controlled growth stage is quickly taken over by the GFN-dominated growth stage (see S4). The rate of the GFN increases; thus, the growing time for the newly formed crystallite at the growth front is shortened with promoted undercooling.

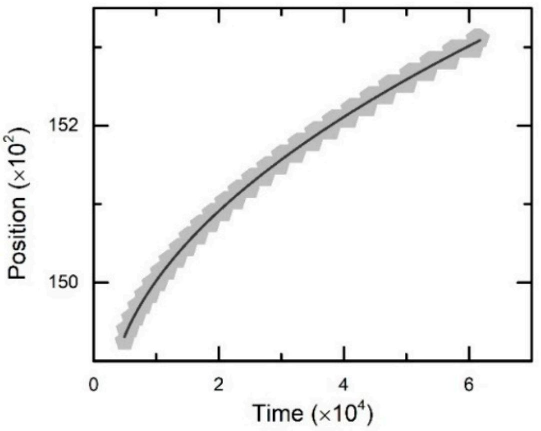

S3. Right tip position versus time for sample II with $\epsilon = 0.1$, where the black solid line is the best fit to relation: $Z = Z_0 + C\sqrt{t - t_0}$, $Z_0 = 14828.578$, $t_0 = 2202.658$, $C = 1.968$.

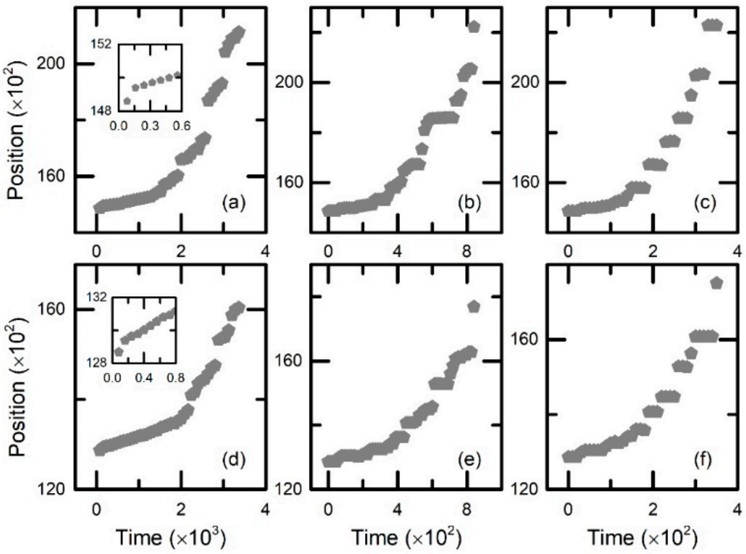

S4. Dendrite tip position versus time for sample II with different undercoolings. (a-c) Right tip position versus time; (d-f) lower tip position versus time. Undercooling parameters for (a) and (d) are $\epsilon = 0.125$, for (b) and (e) are $\epsilon = 0.15$, and for (c) and (f) are $\epsilon = 0.2$. In figure (a) (or d), an enlarged view of the first growth stage is shown in the inset. The growing time for the newly formed crystallite at the growth front is greatly shortened with promoted undercooling.

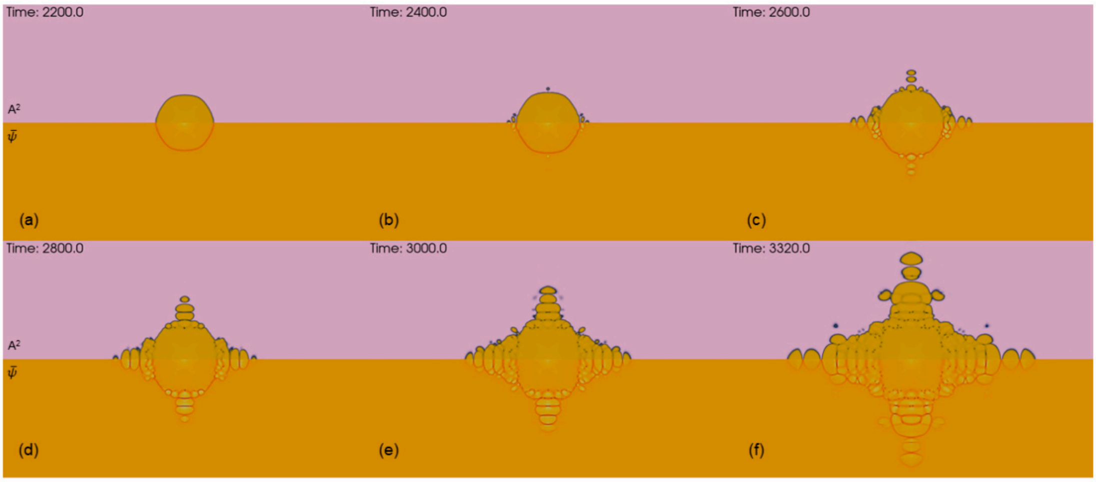

S5. Enlarged contour plots showing the growth processes of the equiaxial dendrite in the main text. The meaning of the colors is the same as in Fig. 9 in the main text.

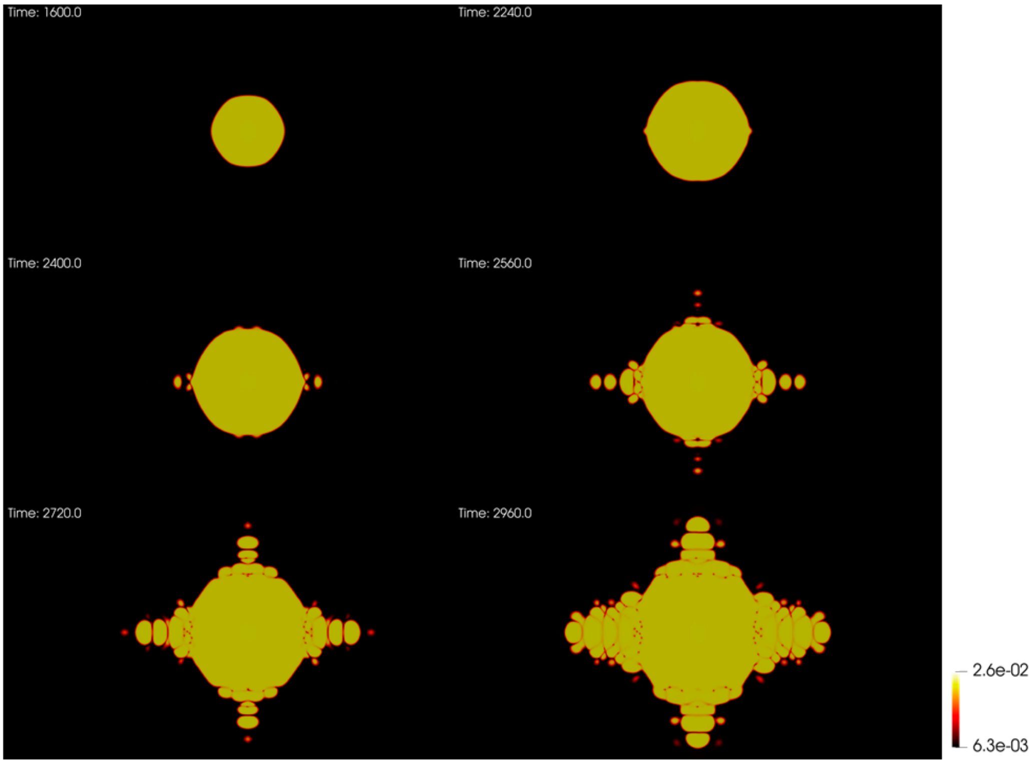

S6. Pattern evolution during the nonequilibrium crystallization of sample II (but with a smaller size, i.e., the same as that shown in Fig. 11 in the main text) under $\epsilon = 0.125$ using PAPFC with M_k = 0.5. The field plotted is A^2. Other conventions are the same as in the main text.

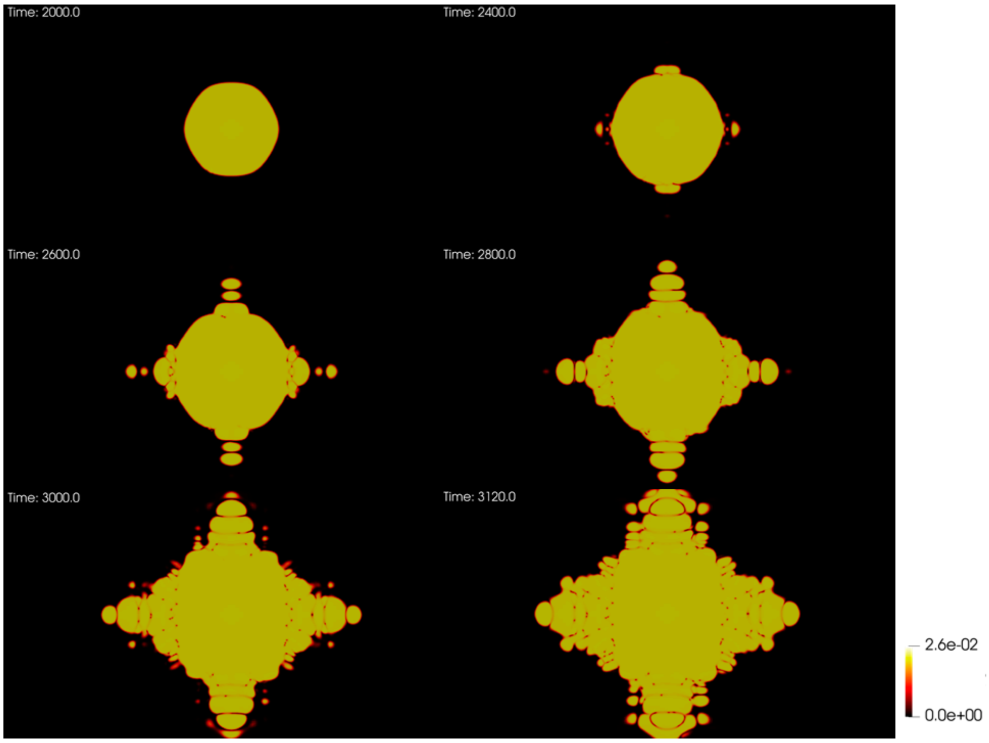

S7. Pattern evolution during the nonequilibrium crystallization of the same sample as that in S6 under $\epsilon = 0.125$ using PAPFC with M_k = 2.0e-3. The field plotted is A^2. Other conventions are the same as in the main text. Compared with the case of large M_k, the beginning time of the first GFN for the small M_k is later. As a result, the central clean grain (without lattice defects) is larger due to the relatively longer growth time. (The growth speed is initially determined by the anisotropic surface energy as well as the mobility parameters for the amplitude and average density. All these factors are the same for the two cases.) Interestingly, the solidification patterns for the two cases are almost the same (See S6 t = 2960 and S7 t = 3000).

References

[1] G. Tegze, L. Gránásy, G. I. Tóth, F. Podmaniczky, A. Jaatinen, T. Ala-Nissila, and T. Pusztai, Physical Review Letters **103**, 035702 (2009).
